# Transcriptome Pathway Analysis of Pathological and Physiological Aldosterone-Producing Human Tissues

**DOI:** 10.1161/HYPERTENSIONAHA.116.08033

**Published:** 2016-11-09

**Authors:** Junhua Zhou, Brian Lam, Sudeshna G. Neogi, Giles S.H. Yeo, Elena A.B. Azizan, Morris J. Brown

**Affiliations:** From the Centre for Clinical Pharmacology, William Harvey Research Institute, Barts and the London School of Medicine & Dentistry, Queen Mary University of London, United Kingdom (J.Z., M.J.B.); Clinical Pharmacology Unit, Department of Medicine, University of Cambridge (J.Z.), University of Cambridge Metabolic Research Laboratories, Wellcome Trust MRC Institute of Metabolic Science (B.L., G.S.H.Y.), Cambridge University Hospitals NHS Foundation Trust (S.G.N.), Addenbrooke's Hospital, United Kingdom; and Department of Medicine, Faculty of Medicine, The National University of Malaysia (UKM) Medical Centre, Kuala Lumpur (E.A.B.A.).

**Keywords:** aldosterone, gene ontology, primary hyperaldosteronism, zona fasciculata, zona glomerulosa

## Abstract

Primary aldosteronism is present in ≈10% of hypertensives. We previously performed a microarray assay on aldosterone-producing adenomas and their paired zona glomerulosa and fasciculata. Confirmation of top genes validated the study design and functional experiments of zona glomerulosa selective genes established the role of the encoded proteins in aldosterone regulation. In this study, we further analyzed our microarray data using AmiGO 2 for gene ontology enrichment and Ingenuity Pathway Analysis to identify potential biological processes and canonical pathways involved in pathological and physiological aldosterone regulation. Genes differentially regulated in aldosterone-producing adenoma and zona glomerulosa were associated with steroid metabolic processes gene ontology terms. Terms related to the Wnt signaling pathway were enriched in zona glomerulosa only. Ingenuity Pathway Analysis showed "NRF2-mediated oxidative stress response pathway" and "LPS (lipopolysaccharide)/IL-1 (interleukin-1)–mediated inhibition of RXR (retinoid X receptor) function" were affected in both aldosterone-producing adenoma and zona glomerulosa with associated genes having up to 21- and 8-fold differences, respectively. Comparing *KCNJ5*-mutant aldosterone-producing adenoma, zona glomerulosa, and zona fasciculata samples with wild-type samples, 138, 56, and 59 genes were differentially expressed, respectively (fold-change >2; *P*<0.05). *ACSS3*, encoding the enzyme that synthesizes acetyl-CoA, was the top gene upregulated in *KCNJ5*-mutant aldosterone-producing adenoma compared with wild-type. *NEFM*, a gene highly upregulated in zona glomerulosa, was upregulated in *KCNJ5* wild-type aldosterone-producing adenomas. *NR4A2*, the transcription factor for aldosterone synthase, was highly expressed in zona fasciculata adjacent to a *KCNJ5*-mutant aldosterone-producing adenoma. Further interrogation of these genes and pathways could potentially provide further insights into the pathology of primary aldosteronism.

## Abstract 2

Supplemental Digital Content is available in the text.

Primary aldosteronism (PA) is estimated to occur in 10% of all hypertensive patients.^1^ Excessive aldosterone in PA patients not only causes hypertension but also brings detrimental effects to multiple systems independent of the blood pressure level.^2,3^ Hypertension (and PA) caused by a unilateral aldosterone-producing adenoma (APA) can be cured or markedly improved by unilateral adrenalectomy. Recent interest in this common, multiple impact, potentially curable syndrome has led to major advances in the molecular understanding of PA. Somatic mutations in the *KCNJ5* gene have been found associated with classical zona fasciculata (ZF)–like APAs, whereas somatic mutations in *ATP1A1, CACNA1D, CTNNB1*, and *ATP2B3* have in some studies been found associated with zona glomerulosa (ZG)–like APAs and aldosterone-producing cell clusters (APCCs).^4–6^ Despite identification of these specific causal mutations, the pathways involved with APA formation in the adrenal remain unclear.

The adrenal ZG is the only physiological site for aldosterone production, whereas the ZF and zona reticularis produce glucocorticoids and sex steroids. Therefore, the 3 adrenocortical zones, and the adrenal medulla (which has a different embryological origin), would have different transcriptome profiles and signaling pathways. Yet previous profiling of APA transcriptomes used as reference whole normal adrenal or whole adrenal cortex without separating the different zones.^7–14^ As an improvement, one study compared a single APA with its adjacent ZG through peeling the capsule (and ZG) from the ZF with a pair of forceps.^15^ However, as human ZG (unlike rodent ZG) is discontinuous, the ZG sample from this study would have included some ZF cells and the adrenal capsule (ie, fibroblast cells). Hence, ZG cells must be discriminated from ZF cells carefully before sampling.

One method of discriminating ZG described by Nishimoto et al^4^ in human adrenal from kidney donors was through immunohistochemistry staining with a *CYP11B2* antisera. However, this method samples only aldosterone-producing ZG cells. To select unbiasedly all ZG cells, we developed a method to differentiate the human ZG from ZF using cresyl violet (previously used by Nishimoto et al^16^ in rats).^17^ This allowed for the comparison of the pathological aldosterone-producing tissue, the APA, to the physiological capable aldosterone-producing tissue, the ZG, and comparison of the ZG with the physiological capable cortisol-producing tissue, the ZF. Comparisons were made between ZG and ZF adjacent to an APA and adjacent to a phaeochromocytoma. This study design accurately identified previously unknown ZG genes, which were functionally shown to regulate aldosterone.^17–19^ Herein, we highlight aberrantly activated or suppressed biological processes and pathways associated with genes differentially regulated between the ZG and APA and analyzed the gene expression profile of adrenals harboring the most common somatic mutation in APAs, the *KCNJ5* mutation.^5,20,21^

## Methods

### Sample Acquisition

Laser capture microdissected samples from the ZG, ZF, and APA were obtained from the adrenals of 14 patients with an APA and 7 patients with a phaeochromocytoma as previously described.^17,22^ Diagnosis of APA was confirmed through immunohistochemistry of *CYP11B2* as detailed in Figure S1 in the online-only Data Supplement. Extracted RNA quality was determined using an Agilent Bioanalyzer 2100 (Pico) and only RNA with a RIN≥7 was used for microarray analysis. Confirmation of selective sample acquisition was done through corticosteroid gene expression profiling (details provided in the Figure S2). In total, paired ZG, ZF, and APA samples were collected from 7 *KCNJ5*-mutant patients, 7 *KCNJ5* wild-type patients, and 7 phaeochromocytoma patients. The clinical features of these patients are detailed in Table S1.

### Microarray Analysis

The 56 RNA samples acquired through laser capture microdissection were loaded onto the Affymetrix Human Gene 1.0 ST Arrays at the Genomics Corelab, Cambridge as previously described.^17^ The microarray result is deposited in the Gene Expression Omnibus database (GSE64957). Validation by quantitative polymerase chain reaction was performed for genes with fold-change >10 and steroidogenic enzymes *CYP11B2*, *CYP11B1*, and *CYP17A1* using standard and validated^23^ custom-made Taqman gene expression assays (AppliedBiosystems). Results were analyzed using the 2^-ΔΔCT^ method with 18S rRNA as the endogenous control.

### Statistical Analysis

The array data were normalized using robust multiarray average and differentially expressed genes were identified as previously described.^17^ Genes with a fold-change >2 and *P*<0.05 were imported into AmiGO 2^24^ for PANTHER (Protein Analysis Through Evolutionary Relationships) over-representation test of GO biological processes (experimental only) using the GO ontology database (release 20150430) and imported into Ingenuity Pathway Analysis (Qiagen Redwood City, CA) for pathway enrichment and top molecular and cellular functions. The *P* value of these analyses was determined based on the number of genes differentially expressed in each process or pathway.

## Results

### APA Versus ZG

#### GO Analysis

To identify the independent alterations of steroidogenesis responsible for PA, gene expression in the pathological aldosterone-producing tissue, the APA, was compared with the reference tissue also differentiated for aldosterone production, the ZG. A total of 277 genes were differentially expressed by >2 fold, of which 104 were upregulated and 173 were downregulated (*P*<0.05).

GO analysis of these 277 genes showed an enrichment of biological processes terms related to genes associated with steroid, alcohol, and organic hydroxy compound metabolic processes, genes associated with cell migration and angiogenesis, and genes associated with the regulation of body fluid levels (Table 1).

**Table 1.**
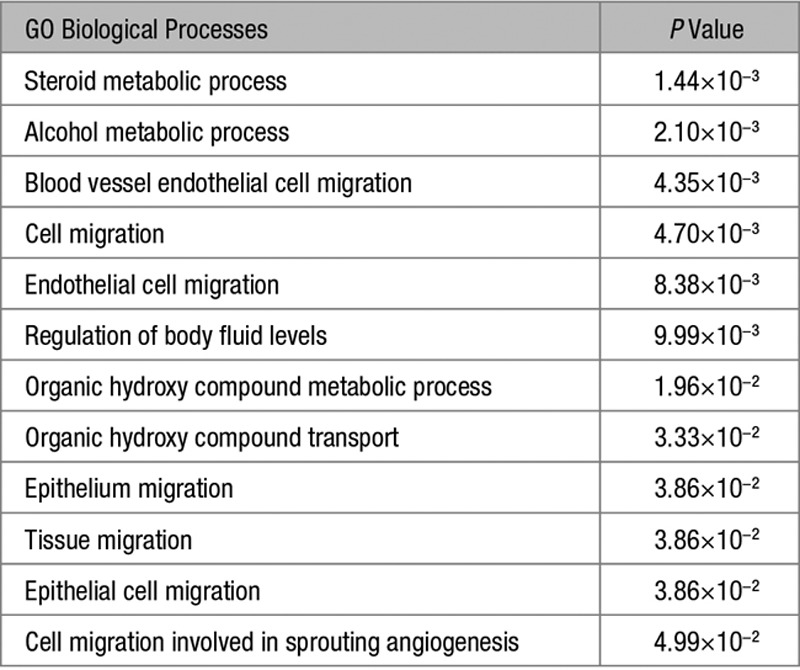
GO Terms of Biological Processes ≥5-Fold-Enriched in Aldosterone-Producing Adenoma Compared With Zona Glomerulosa

Genes that were associated with steroid, alcohol, and organic hydroxy compound metabolic processes that were upregulated in APA versus ZG were downregulated in ZG versus ZF, and vice versa (Table S2). A similar trend was seen in genes associated with cell migration and angiogenesis and genes associated with regulation of body fluid levels (Tables S3 and S4). The top 10 genes that were downregulated in APA versus ZG overlapped with the top 10 genes upregulated in ZG versus ZF (ie, *LGR5*, *ANO4*, *VCAN*, and *SFRP4* as shown in Tables S5 and S6). Further analysis found 108 of the 173 genes that were downregulated in APAs versus ZG were upregulated in ZG versus ZF.

#### Pathway Analysis

The top 5 canonical pathways enriched in APA versus ZG were NRF2-mediated oxidative stress response, complement system, lipopolysaccharide (LPS)/interleukin-1 (IL-1)–mediated inhibition of retinoid X receptor (RXR) function, glucocorticoid biosynthesis, and androgen biosynthesis (Table 2).

**Table 2.**
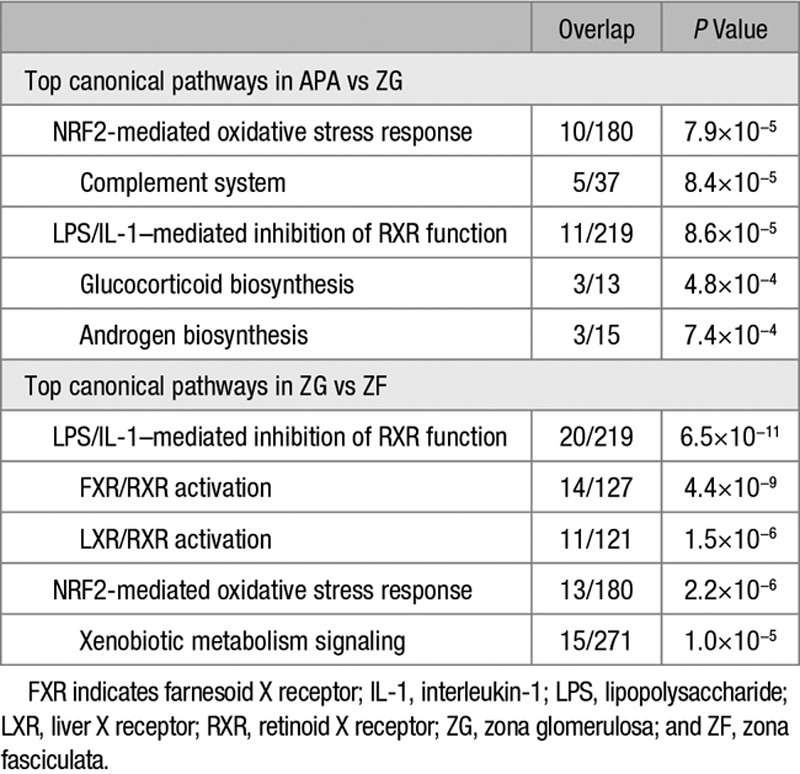
Top 5 Canonical Pathways Associated With Genes Differentially Regulated in APA Compared With ZG and ZG Compared With ZF

The top molecular and cellular functions in APAs (versus ZG), cell death and survival, lipid metabolism, and small-molecule biochemistry, overlapped with the top molecular and cellular functions enriched in ZG (versus ZF; Table 3). No overlap was seen for cellular growth and proliferation and cellular movement. The molecular and cellular functions, vitamin and mineral metabolism, and molecular transport were uniquely enriched in genes differentially regulated in ZG versus ZF.

**Table 3.**
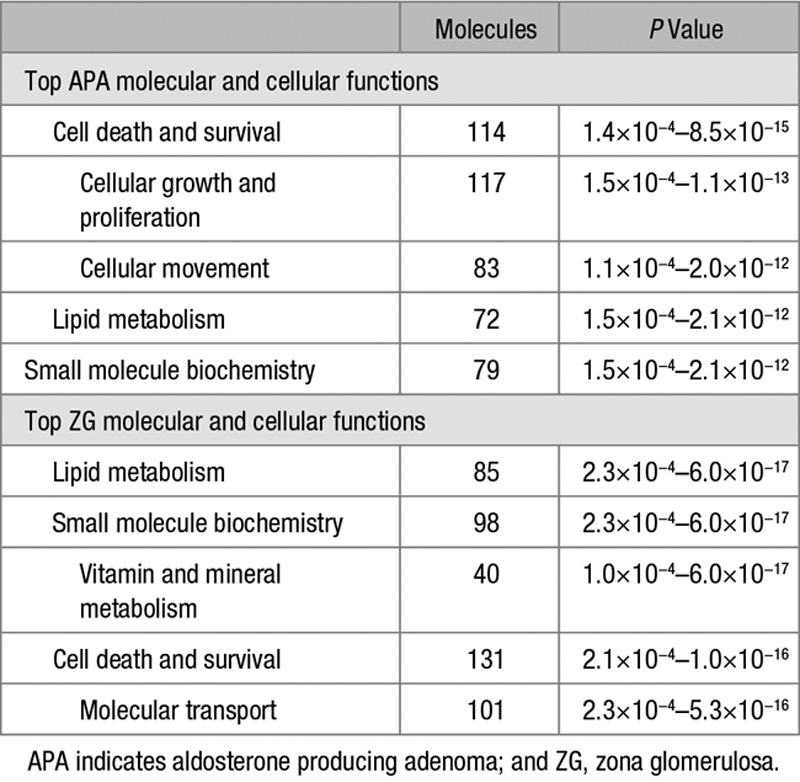
Top 5 Molecular and Cellular Functions Enriched in APA (Compared With ZG) and in ZG (Compared With Zona Fasciculata)

APAs were also separately compared with ZG samples adjacent to a phaeochromocytoma only. Six of the top 10 genes (up- and downregulated) and all the top 5 molecular and cellular functions were the same as identified when comparing APA with all ZG samples (Tables S7 and S8, respectively). Similarly, LPS/IL-1–mediated inhibition of RXR function was highlighted as 1 of the top 5 canonical pathways regardless of whether APA was compared with all ZG samples or phaeochromocytoma samples only (Table 2; Table S9).

### ZG Versus ZF

#### GO Analysis

A total of 334 genes were differentially expressed by >2-fold when comparing all ZG samples with all ZF samples, of which 213 were upregulated and 121 were downregulated in ZG (*P*<0.05). GO analysis of these genes showed an enrichment of terms related to steroid, cholesterol, lipid, lipoprotein, alcohol biological processes, and terms related to Wnt signaling (Table 4). Similar to genes associated with steroid, organic hydroxy compound, and alcohol-related processes (Table S2), 8 genes associated with the Wnt signaling pathway that were upregulated in ZG versus ZF were downregulated in APA versus ZG (Table S10).

**Table 4.**
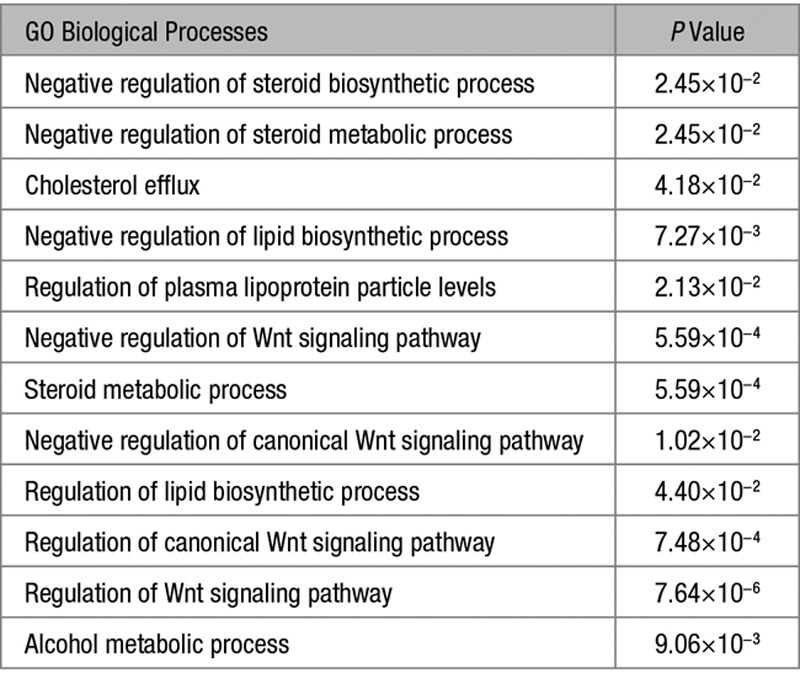
GO Terms of Biological Processes ≥5-Fold Enriched in Zona Glomerulosa Compared With Zona Fasciculata

The enrichment of biological processes terms related to genes associated with steroid, cholesterol, lipid, lipoprotein, alcohol-related processes, and genes associated with the Wnt signaling pathway remained true irrespective of whether the comparison was made separately for ZG adjacent to an APA or a phaeochromocytoma (Tables S10–S13). In agreement, 165 of the genes upregulated and 76 of the genes downregulated in ZG were the same when comparing all ZG samples with all ZF samples or when comparing samples from phaeochromocytoma patients only. Of the top 10 genes upregulated in all ZG samples, 8 remained the same when only comparing samples from pheochromocytoma patients (Table S6). All of the genes, but 2 (*ITGA3* and *GRB10*), associated with the Wnt signaling pathway were upregulated in ZG versus ZF samples, irrespective of whether the comparison was made separately for ZG adjacent to an APA or a phaeochromocytoma (Tables S10 and S11).

However, some genes associated with steroid, organic hydroxy compound, and alcohol-related processes that were upregulated in ZG samples from phaeochromocytoma patients (eg, *CYP11B2*, *CYP39A1, PLA2G1B*, and *SULT1E1*), were not significantly upregulated in ZG samples from adrenals with an APA (Table S12). Comparing the biological processes enriched in ZG versus ZF, we found that negative regulation of steroid biosynthetic process, negative regulation of steroid metabolic process, cholesterol efflux, negative regulation of lipid biosynthetic process, and regulation of plasma lipoprotein particle levels did not occur when only comparing samples adjacent to a phaeochromocytoma (Table 4; Table S13).

#### Pathway Analysis

Pathway analysis of genes differentially regulated in ZG (versus ZF) highlighted the same canonical pathways as that with APAs versus ZG: NRF2-mediated oxidative stress response and LPS/IL-1–mediated inhibition of RXR function (Table 3). FXR/RXR activation, LXR/RXR activation, and xenobiotic metabolism signaling pathways were uniquely affected in ZG (versus ZF). LPS/IL-1–mediated inhibition of RXR function remained the top canonical pathway affected even when only interrogating ZG samples adjacent to a phaeochromocytoma (Table S14).

**Figure 1.**
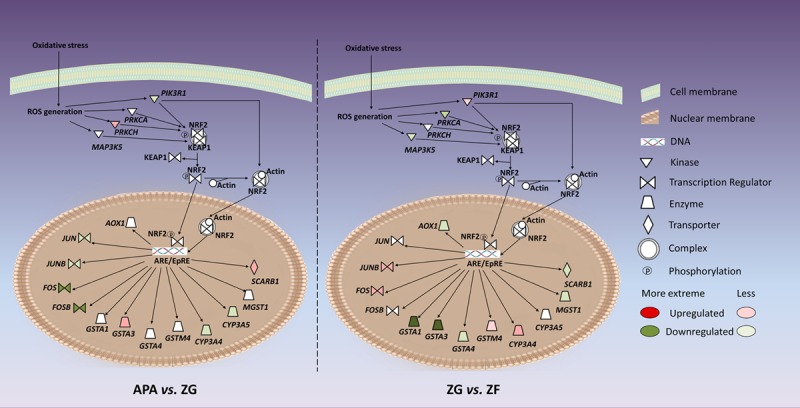
Genes associated with the NRF2-mediated oxidative stress response pathway that were differentially regulated in aldosterone-producing adenoma (APA) vs zona glomerulosa (ZG) and ZG vs zona fasciculata (ZF).

Thirteen genes involved in the NRF2-mediated oxidative stress response canonical pathway were differentially expressed in ZG versus ZF, of which 10 were also differentially expressed in APA versus ZG (Figure 1; details in Table S15). The most differentially expressed in APA versus ZG were FBJ murine osteosarcoma viral oncogene homolog B (*FOSB*) and FBJ murine osteosarcoma viral oncogene homolog (*FOS*), both with an ≈6-fold-change. The most differentially expressed in ZG versus ZF were glutathione S-transferase alpha 1 (*GSTA1*) and glutathione S-transferase alpha 3 (*GSTA3*), with an ≈21-fold-change and a 9-fold-change, respectively.

**Figure 2.**
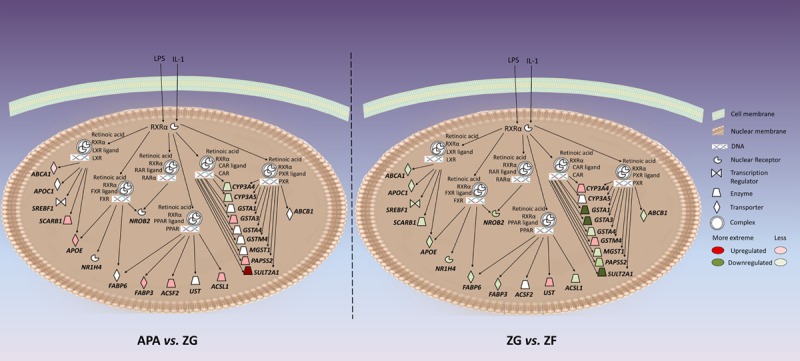
Genes associated with the lipopolysaccharide (LPS)/interleukin-1 (IL-1)–mediated inhibition of retinoid X receptor (RXR) function pathway that were differentially regulated in aldosterone-producing adenoma (APA) vs zona glomerulosa (ZG) and ZG vs zona fasciculata (ZF). CAR indicates constitutive androstane receptor; and RAR indicates retinoic acid receptor.

Twenty genes involved in the LPS/IL-1–mediated inhibition of RXR function canonical pathway were differentially expressed in ZG versus ZF, of which 11 were also differentially expressed in APA versus ZG (Figure 2; details in Table S16). Among the most differentially expressed genes in this pathway were once again *GSTA1* and *GSTA3*, and sulfotransferase family 2A member 1 (*SULT2A1*),which was 8.21-fold downregulated in ZG compared with ZF and the most upregulated (11.08-fold) in APA compared with ZG (Table S16).

### KCNJ5 Genotypes

Comparing *KCNJ5* mutation status, 138 genes were differentially expressed in mutant APAs versus *KCNJ5* wild-type APAs, of which 61 genes were upregulated (fold-change >2; *P*<0.05). *ACSS3*, encoding the enzyme that synthesizes acetyl-CoA, an important precursor of cholesterol, was the top gene upregulated in *KCNJ5*-mutant APAs compared with wild-type ones. Interestingly, *NEFM* (encoding the neurofilament triplet M protein), a gene previously validated by quantitative polymerase chain reaction to be almost 200-fold upregulated in ZG (versus ZF),^17^ was upregulated in *KCNJ5* wild-type APAs (versus mutant APAs; Tables S6 and S17).

Comparing ZG samples adjacent to a *KCNJ5*-mutant APA or a wild-type APA, 56 genes were differentially expressed of which 30 genes were upregulated (fold-change >2; *P*<0.05). The top 10 differentially regulated genes are shown in Table S18.

Comparing ZF samples in a similar manner, 59 genes were differentially expressed of which 38 genes were upregulated in ZF samples adjacent to a *KCNJ5*-mutant APA (fold-change >2; *P*<0.05). Surprisingly, *NR4A2* (the transcription factor of *CYP11B2*) was found to be the most upregulated gene (Table S19). Validation by quantitative polymerase chain reaction showed >50-fold higher *NR4A2* mRNA expression in ZF samples adjacent to a *KCNJ5*-mutant APA than those adjacent to *KCNJ5* wild-type APA (n=6; *P*=0.007).

Molecular and cell function analysis suggested some differences between APAs with different *KCNJ5* genotypes, and in the ZF samples adjacent to them (Table S20). In contrast, little difference (<10 genes affected per function) was found between ZG samples adjacent to APAs with different *KCNJ5* genotypes (Table S20). Similarly, canonical pathway analysis found little difference between samples from patients with different genotype, all with a *P*>1.0×10^–3^ (Table S21). No significant enrichments of GO biological processes terms were found.

## Discussion

This study performed GO and pathway analyses on genes expressed in APAs and ZG—one a pathological aldosterone-producing tissue and the other a physiological aldosterone-producing tissue. Previous transcriptomic studies comparing APAs with normal adrenal glands unavoidably selects ZF, the major component of the adrenal cortex, as their reference samples.^7–13,23,25–28^ As the ZF is not physiologically capable of producing aldosterone, such comparisons would be difficult to interpret when seeking to understand the pathological mechanism of autonomous aldosterone production in APAs. Despite the disadvantage of previous transcriptomic studies, many pathways associated with aldosterone regulation have been identified to be aberrantly activated or suppressed in APAs: the Wnt/β-catenin signaling pathway, the PI3K/AKT signaling pathway, the MAPK signaling pathway, and the calcium signaling pathway.^12,13,15,29^ Genes found differentially expressed in the APA compared with normal adrenal includes G-protein–coupled hormone receptors such as *GnRH*, *GPR37*, *GRM3*, *LH*, *AVP*, and *HTR4*^7,8,10,11,30^ and steroid enzymes such as *CYP11B2*, *CYP11B1*, *CYP17A1*, and *StAR*. In our comparison between APA and ZG, most of the top genes were not reported before to be associated with PA. Similarly, the most significant pathways enriched in both APAs (versus ZG) and ZG (versus ZF), the NRF2-mediated oxidative stress response and the LPS/IL-1–mediated inhibition of RXR function pathways, had not been highlighted in past transcriptomic studies. We postulate the differences seen in our findings are because of our differentiation of the adrenal cortex transcriptomes to transcriptomes from the ZG and transcriptomes from the ZF.

NRF2-mediated oxidative stress response pathway is an important pathway for cell survival as severe oxidative stress can activate apoptosis and necrosis. This pathway has been associated with oxidative stress-mediated diseases such as cancer, neurodegenerative, cardiovascular and pulmonary complications.^31^ The pathway involves binding of NRF2 to antioxidant response elements, which activates their transcription. The role of this pathway in PA is supported by recent findings of increased oxidative stress in PA patients measured by high NADPH oxidase (nicotinamide adenine dinucleotide phosphate–oxidase; Nox2-dp) plasma levels and urinary isoprostanes preadrenalectomy^32^ and the previous finding in a single APA of increased expression of oxidative stress–related proteins.^33^ As oxidative stress mediates genotoxic effects,^34^ and oxidative damage is postulated to occur in the adrenal,^35^ the findings of >50% of APAs pathological aldosterone production due to a somatic mutation^5,20,21^ collectively suggest that oxidative stress could be involved in mediating DNA damage in driver genes resulting in the pathological corticosteroid production.

Of the 2 overlapping pathways, only LPS/IL-1–mediated inhibition of RXR (retinoid X receptor) function pathway remained affected when only comparing ZG samples adjacent to a phaeochromocytoma (Tables S9 and S14). RXR is a ligand-dependent nuclear receptor that forms a complex with other ligand-dependent nuclear receptors and affects transcriptional receptors.^36^ LPS and IL-1 can trigger the inhibition of this transcriptional cassette.^37^ It has been reported that the reduction of proteins related to the LPS/IL-1–mediated inhibition of RXR function pathway can lead to impaired metabolism, transport or biosynthesis of lipid, cholesterol, bile acid, and xenobiotics.^38,39^ In APAs, genes related to this pathway were mainly upregulated, suggesting perhaps a role in the metabolism, transport, and biosynthesis of cholesterol, a key precursor of aldosterone. On the contrary, genes associated with this pathway were downregulated in ZG (Table S16), suggesting decrease of corticosteroid synthesis, which is in agreement with our previous finding of ZG selective genes regulating aldosterone negatively and the low patchy staining of CYP11B2 in ZG.^17–19^

One pathway that had already been associated previously with aldosterone regulation is the Wnt signaling pathway. A transcriptomic study in rodents found 3 Wnt-related proteins enriched in rat ZG: *CPZ*, *GPC3*, and *WNT4*.^40^ We similarly found an enrichment of GO terms related to the Wnt signaling pathway when comparing human ZG with ZF. However, investigation in human adrenal cells found *LGR5* (the most upregulated Wnt-related ZG-gene in humans) to reduce aldosterone production and *CYP11B2* mRNA expression, increase apoptosis, and reduce proliferation,^18^ whereas *WNT4* deficiency in mice reduced aldosterone production and *CYP11B2* mRNA expression,^41^ and constitutive β-catenin activation caused the development of PA and promoted malignancy.^42^ Interestingly, *LGR5* has also been observed to be downregulated in APCCs compared with ZG in human adrenals from kidney donors.^4^

The opposite findings from humans and rodents could be without significance and ascribed to random species variation. In agreement, little overlap was found between the most upregulated genes in ZG from the rodent study^16,40^ and our human study (only 3 of the top 50: *RGS4*, *ATP10A*, and *PDE2A*),^17^ and similarly different potassium channels were found to dominate the ZG K^+^ currents in the 2 species.^43^ In addition, we speculate that some of the species variation could be a result of human’s chronic exposure to salt. We hypothesize, in chronic salt environment, that the ZG cells in human adrenals undergo apoptosis when not producing aldosterone. This hypothesis is based on (1) CYP11B2^−/−^ mice, where ZG cells migrate and apoptose,^44^ (2) the disappearance from most of human ZG of CYP11B2, which became apparent with selective antisera,^45^ (3) well-known suppression of renin–aldosterone production by salt excess, (4) our own apoptosis data in human ZG.^18^ Thus, rodents may not be ideal models for PA (or even physiological production of aldosterone), and a human model, such as our microarray study, is needed to specifically understand the regulation of aldosterone in humans.

Our earlier microarray comparing *KCNJ5*-mutant APAs with *ATP1A1*- or *CACNA1D*-mutant APAs (all wild-type for *KCNJ5*) identified 43 genes differentially regulated (fold-change >2; false discovery rate <0.05).^6^ In our current microarray, we found the difference between *KCNJ5*-mutant and *KCNJ5* wild-type APAs to be much smaller. Although 138 genes were differentially regulated (fold-change >2; *P*<0.05), none passed the false discovery rate <0.05 threshold. Nevertheless, we did once again find *MYOM1*, *BEX1*, *VPREB3*, *NETO2*, and *PTPRZ1* to be differentially regulated (fold-change >2; *P*<0.05), as previously published when comparing *CACNA1D*/*ATP1A1* mutant APAs with *KCNJ5*-mutant APAs (Table S14).^6^ Thus, although tissues with different genotypes have similar transcriptome profiles, some key marginal differences do exist between differing *KCNJ5* genotypes. For example, *ACSS3* was the top upregulated gene in *KCNJ5*-mutant APA. The encoded protein ligates acetate and CoA to make acetyl-CoA, the sole carbon source and precursor for both fatty acid and cholesterol biosynthesis in mammalian cells. Therefore, it could potentially be the reason for the high aldosterone production seen in patients with *KCNJ5*-mutant APAs despite the low expression of *CYP11B2* in the adenoma.^46^

Another key difference was *NEFM*, a highly selective ZG-gene that was upregulated in *KCNJ5* wild-type APAs compared with mutant APAs (Tables S6 and S14). We had previously reported another ZG selective gene, nephronectin (*NPNT*), to be significantly upregulated in *ATP1A1*- or *CACNA1D*-mutant APAs compared with *KCNJ5*-mutant APAs.^6^ This may be seen as an unbiased support for our current paradigm that *KCNJ5*-mutant APAs (unlike *ATP1A1* and *CACNA1D*-mutant APAs) are ZF like and may have arisen from the cortisol-producing ZF instead of ZG.^22^ In further support of this proposition, *NR4A2*, the transcription factor for aldosterone production, was the most highly expressed gene in ZF adjacent to a *KCNJ5*-mutant APA (versus wild-type ZF; Table S19). However, additional studies are warranted in interrogating the origin and formation of these APAs especially because APCCs, whom some propose are precursors of APAs, can be present in ZF, and APCCs have a high rate of mutation in *CACNA1D* and *ATP1A1* but not in *KCNJ5*.^4^

The strengths of the study of comparing APA samples with adjacent ZG are also the limitations of this study. These ZG samples would have aldosterone production suppressed by the paracrine effect of the APA. Therefore, we have supplemented our data with APA versus phaeochromocytoma although these also may have their own paracrine effect of the adenoma.^47^ Considering that the reported difference of CYP11B2 expression in normal ZG versus ZF, harvested from healthy renal transplantation donors, is also only about 2-fold (as seen by the microarray results for the four ZG samples),^4^ it seems that the aldosterone production in human ZG, whether normal or adjacent to a pheochromocytoma or an APA, is suppressed. Furthermore, our method of undiscriminatory sampling of ZG cells prevents enrichment of populations of aldosterone-producing ZG cells or knowledge of samples containing APCCs. We are reassured, however, that a quick comparison of the top ZG genes (eg, *LGR5*) suggests that the transcriptomes of our ZG samples are different from APCCs and more similar to ZG samples acquired from the adrenal of kidney donors.^4^

## Perspectives

We have identified a list of novel genes and canonical pathways enriched in pathological and physiological aldosterone-producing human tissues. Through exploring the role of these highlighted genes and pathways on aldosterone production, the molecular mechanism of APA formation and pathology could potentially be unraveled further.

## Acknowledgments

We thank Ms Ada E.D. Teo for her assistance in proof-reading the article.

## Sources of Funding

The work was funded by a National Institute for Health Research (NIHR) Senior Investigator Award (NF-SI-0512-10052) to M.J. Brown and the NIHR Cambridge Biomedical Research Centre (Cardiovascular). E.A.B. Azizan was supported by The National University of Malaysia Young Investigator Award (GGPM-2015-012). J. Zhou was supported by The Cambridge International Trust and the Sun Hung Kai Properties–Kwoks’ Foundation.

## Disclosures

None.

## Supplementary Material

## Novelty and Significance

### What Is New?

- We performed gene ontology and pathway analysis on transcriptome profiles of laser capture microdissected paired human aldosterone-producing adenoma (APA), zona glomerulosa (ZG), and zona fasciculata samples.

### What Is Relevant?

- We identified a list of novel genes, biological processes, and canonical pathways that may be involved with APA pathology and ZG cell functions. We also investigated the effect of the most common somatic mutation (*KCNJ5*) in APA, ZG, and zona fasciculata tissues, respectively.

### Summary

Comparison of transcriptomes from an APA, a pathological aldosterone-producing tissue, and ZG, a physiological aldosterone-producing tissue, identified steroid metabolic processes, Wnt signaling pathway, NRF2-mediated oxidative stress response pathway, LPS (lipopolysaccharide)/IL-1 (interleukin-1)–mediated inhibition of RXR function to play a role in aldosterone regulation. Comparison of tissue samples by the presence of a *KCNJ5*-mutant APA found (1) *ACSS3*, encoding the enzyme that synthesizes acetyl-CoA, to be upregulated in *KCNJ5*-mutant APAs; (2) *NEFM*, a gene highly upregulated in ZG, to be upregulated in *KCNJ5* wild-type APAs; and (3) *NR4A2*, the transcription factor for aldosterone synthase, to be highly expressed in zona fasciculata adjacent to a *KCNJ5*-mutant APA. These genes and pathways could potentially provide further insights into the pathology of PA.
