# Supplementary material for: Transcriptome Pathway Analysis of Pathological and Physiological Aldosterone-Producing Human Tissues
Source: Hypertension. 2016 Nov 9;68(6):1424–31. doi: 10.1161/HYPERTENSIONAHA.116.08033 (PMC5100803; doi:10.1161/HYPERTENSIONAHA.116.08033)
Supplement: Supplementary file 1 [file hyp-68-1424-s001.docx]

**TRANSCRIPTOME PATHWAY ANALYSIS OF PATHOLOGICAL AND PHYSIOLOGICAL ALDOSTERONE-PRODUCING HUMAN TISSUES**

Junhua Zhou*^1,5^, Brian Lam*^2^, Sudeshna G. Neogi^3^, Giles S.H Yeo^2^, Elena A.B. Azizan*^4^, Morris J. Brown*^5^

^1^Clinical Pharmacology Unit, Department of Medicine, University of Cambridge, Addenbrooke’s Hospital, Cambridge, United Kingdom;

^2^University of Cambridge Metabolic Research Laboratories, Wellcome Trust-MRC Institute of Metabolic Science, Addenbrooke's Hospital, Hills Road, Cambridge, United Kingdom.

^3^Cambridge University Hospitals NHS Foundation Trust, Addenbrooke’s Hospital, Cambridge, United Kingdom.

^4^Department of Medicine, Faculty of Medicine, The National University of Malaysia (UKM) Medical Centre, Kuala Lumpur, Malaysia.

^5^Barts Heart Centre, William Harvey Research Institute, Queen Mary University London, United Kingdom.

*These authors contributed equally to this work.

This PDF contains: Supplementary Materials and Methods

Table S1-S21

Figure S1-S2

**Supplementary Materials and Methods**

**Diagnosis of APA.** Immunohistochemistry of CYP11B2 was performed either manually using the custom-made primary antibody gifted by Dr Celso E Gomez-Sanchez or automatically (Bond-III system, Leica Biosystems) using our own custom-made primary antibody that had been affinity purified against the immunizing antigen and subsequently purified against a CYP11B1 antigen column to remove any cross-reacting antibodies. Immunohistochemistry was performed on formalin-fixed, paraffin-embedded adrenal sections (4 μm). Negative controls, whereby primary antibodies were omitted, resulted in a complete absence of staining. Images were captured using a standard bright field microscope, U-TV1-X digital camera and the CellD software (Olympus Ltd, UK).

**Table S1.** Summary of the clinical features of the patients with *KCNJ5* mutant or wild-type APAs involved in this study.

| *KCNJ5* Genotype | Mutant | Wild-type | *P*-value |
| --- | --- | --- | --- |
| Age at surgery | 46.6 ±5.1 | 44.6 ±3.4 | >0.1 |
| Sex (M/F) | 2/5 | 5/2 |  |
| Adrenal side (L/R) | 1/6 | 4/3 |  |
| APA diameter (mm) | 22.4 ±1.2 | 14.5 ±2.3 | 0.01 |
| % of ZG-like cells | 22.9 ±8.4 | 56.7 ±13.5 | 0.04 |
| Pre-adrenalectomy |  |  |  |
| SBP (mmHg) | 144.4 ±4.7 | 170.1 ±13.2 | 0.07 |
| DBP (mmHg) | 90.4 ±8.1 | 104.0 ±8.2 | >0.1 |
| Serum K^+^ (mmol/L) | 3.3 ±0.2 | 3.8 ±0.2 | 0.10 |
| Plasma aldo (pmol/L) | 1029.0 ±389.3 | 1080.2 ±296.1 | >0.1 |
| Plasma renin (mU/L) | 3.6 ±1.0 | 10.0 ±4.8 | >0.1 |
| Post-adrenalectomy |  |  |  |
| SBP (mmHg) | 114.9 ±6.2 | 130.4 ±3.8 | 0.04 |
| DBP (mmHg) | 75.9 ±3.8 | 81.1 ±4.0 | >0.1 |
| Serum K^+^ (mmol/L) | 4.4 ±0.2 | 4.4 ±0.1 | >0.1 |
| Plasma aldo (pmol/L) | 135.6 ±25.0 | 146.4 ±31.8 | >0.1 |
| Plasma renin (mU/L) | 30.0 ±11.5 | 60.4 ±25.7 | >0.1 |

**Table S2.** Genes associated with steroid, alcohol, and organic hydroxy compound related metabolic processes that were differentially regulated in aldosterone-producing adenoma (APA) (compared to zona glomerulosa (ZG)) and in ZG (*vs.* zona fasciculata (ZF)).

| Gene symbol | Protein name | Fold-change (APA *vs.* ZG) | *P*-value  (APA *vs.* ZG) | Fold-change  (ZG *vs.* ZF) | *P*-value  (ZG *vs.* ZF) |
| --- | --- | --- | --- | --- | --- |
| *CYP11B2* | Cytochrome P450 11B2 | 5.63 | 4.84 x 10^-7^ | n.s. |  |
| *CYP11A1* | Cholesterol side-chain cleavage enzyme | 3.13 | 8.79 x 10^-11^ | -4.31 | 5.39 x 10^-16^ |
| *SCARB1* | Scavenger receptor class B member 1 | 2.56 | 2.51 x 10^-10^ | -3.05 | 3.46 x 10^-14^ |
| *HSD17B14* | 17-beta-hydroxysteroid dehydrogenase 14 | 2.54 | 1.96x 10^-4^ | -2.11 | 1.27 x 10^-10^ |
| *LDLR* | Low-density lipoprotein receptor | 2.53 | 3.06 x 10^-4^ | n.s. |  |
| *SORD* | Sorbitol dehydrogenase | 2.40 | 8.05 x 10^-4^ | -2.53 | 1.05 x 10^-4^ |
| *NPC1* | Niemann-Pick C1 protein | 2.35 | 2.14 x 10^-6^ | -3.09 | 2.18 x 10^-10^ |
| *APOE* | Apolipoprotein E | 2.13 | 4.45 x 10^-4^ | -3.05 | 1.01 x 10^-7^ |
| *ABCA1* | ATP-binding cassette sub-family A member 1 | 2.05 | 3.07 x 10^-9^ | -2.39 | 3.65 x 10^-13^ |
| *FDX1* | Adrenodoxin, mitochondrial | 2.03 | 7.00 x 10^-9^ | n.s. |  |
| *CYP17A1* | Steroid 17-alpha-hydroxylase/17,20 lyase | 2.02 | 8.74 x 10^-4^ | -3.34 | 1.27 x 10^-8^ |
| *DHCR24* | Delta(24)-sterol reductase | 2.01 | 1.42 x 10^-9^ | -2.34 | 1.08 x 10^-13^ |
| *ADH1B* | Alcohol dehydrogenase 1B | -2.13 | 1.85 x 10^-5^ | n.s. |  |
| *AQP1* | Aquaporin-1 | -2.50 | 3.06 x 10^-5^ | 2.77 | 6.68 x 10^-7^ |
| *CYP3A4* | Cytochrome P450 3A4 | -3.38 | 1.10 x 10^-7^ | 3.10 | 4.93 x 10^-8^ |
| *RBP4* | Retinol-binding protein 4 | -4.74 | 6.40 x 10^-7^ | 5.03 | 3.79 x 10^-7^ |

n.s., not significant. APOE, *CYP11A1*, *CYP11B2*, *CYP1B1*, *CYP3A4*, *DHCR24*, and *DHCR7* were associated with all 3 GO terms.

**Table S3.** Genes associated with cell migration and angiogenesis that were differentially regulated in aldosterone-producing adenoma (APA) (compared to zona glomerulosa (ZG)) and in ZG (*vs.* zona fasciculata (ZF)).

| Gene symbol | Protein name | Fold-change (APA *vs.* ZG) | *P*-value  (APA *vs.* ZG) | Fold-change (ZG *vs.* ZF) | *P*-value (ZG *vs.* ZF) |
| --- | --- | --- | --- | --- | --- |
| *DPP4* | Dipeptidyl peptidase 4 | 2.32 | 9.72 x 10^-4^ | n.s. |  |
| *EMP2* | Epithelial membrane protein 2 | -2.11 | 2.29 x 10^-3^ | 2.05 | 1.16 x 10^-3^ |
| *PDGFRB* | Platelet-derived growth factor receptor beta | -2.18 | 7.55 x 10^-7^ | n.s. |  |
| *LAMB1* | Laminin subunit beta-1 | -2.24 | 1.74 x 10^-4^ | 3.84 | 7.83 x 10^-10^ |
| *SLIT2* | Slit homolog 2 protein | -2.40 | 5.68 x 10^-8^ | 3.27 | 4.79 x 10^-13^ |
| *HGF* | Hepatocyte growth factor | -2.57 | 4.35 x 10^-6^ | n.s. |  |
| *EFNB2* | Ephrin-B2 | -2.62 | 3.75 x 10^-8^ | n.s. |  |
| *NR4A1* | Nuclear receptor subfamily 4 group A member 1 | -2.77 | 3.01 x 10^-4^ | 4.05 | 2.47 x 10^-7^ |
| *PDGFRA* | Platelet-derived growth factor receptor alpha | -2.78 | 2.31 x 10^-9^ | n.s. |  |
| *BAMBI* | BMP and activin membrane-bound inhibitor homolog | -3.28 | 1.12 x 10^-4^ | 2.75 | 2.16 x 10^-4^ |
| *CCL21* | C-C motif chemokine 21 | -4.18 | 2.09 x 10^-4^ | n.s. |  |
| *EGR3* | Early growth response protein 3 | -4.57 | 4.20 x 10^-7^ | 4.98 | 9.93 x 10^-9^ |
| *CYR61* | Protein CYR61 | -6.98 | 5.51 x 10^-7^ | 3.31 | 2.56 x 10^-4^ |
| *VCAN* | Versican core protein | -8.08 | 6.72 x 10^-9^ | 14.34 | 1.85 x 10^-13^ |

*n.s., not significant.

**Table S4.** Genes associated with regulation of body fluid levels that were differentially regulated in aldosterone-producing adenoma (APA) (compared to zona glomerulosa (ZG)) and in ZG (*vs.* zona fasciculata (ZF)).

| Gene symbol | Protein name | Fold-change  (APA *vs.* ZG) | *P*-value  (APA *vs.* ZG) | Fold-change  (ZG *vs.* ZF) | *P*-value  (ZG *vs.* ZF) |
| --- | --- | --- | --- | --- | --- |
| *CYP11B2* | Cytochrome P450 11B2, mitochondrial | 5.63 | 4.84 x 10^-7^ | n.s. |  |
| *CLDN1* | Claudin-1 | -2.05 | 1.16 x 10^-5^ | 2.83 | 2.30 x 10^-10^ |
| *EMP2* | Epithelial membrane protein 2 | -2.11 | 2.30 x 10^-3^ | 2.05 | 1.16 x 10^-3^ |
| *FBLN1* | Fibulin-1 | -2.17 | 4.36 x 10^-8^ | 2.07 | 1.29 x 10^-8^ |
| *AQP1* | Aquaporin-1 | -2.50 | 3.06 x 10^-5^ | 2.77 | 6.68 x 10^-7^ |
| *PDGFRA* | Platelet-derived growth factor receptor alpha | -2.78 | 2.31 x 10^-9^ | n.s. |  |
| *HBB* | Hemoglobin subunit beta | -2.94 | 2.31 x 10^-3^ | n.s. |  |
| *CSRP1* | Cysteine and glycine-rich protein 1 | -2.97 | 2.34 x 10^-6^ | 2.38 | 1.96 x 10^-5^ |

*n.s., not significant.

**Table S5.** Top 10 genes up-and down-regulated in aldosterone-producing adenoma (APA) compared with zona glomerulosa (ZG).

| Top genes up-regulated | Fold-change | *P*-value |
| --- | --- | --- |
| *SULT2A1* | 11.08 | 1.8 x 10^-16^ |
| *MC2R* | 10.19 | 2.1 x 10^-9^ |
| *SLC37A2* | 5.66 | 5.1 x 10^-7^ |
| *CYP11B2* | 5.63 | 4.8 x 10^-7^ |
| *LRRC39* | 5.32 | 1.1 x 10^-10^ |
| *PRLR* | 4.48 | 4.2 x 10^-12^ |
| *GSTA3* | 4.01 | 3.8 x 10^-8^ |
| *SYTL5* | 4.00 | 1.4 x 10^-8^ |
| *RRAGD* | 3.96 | 7.7 x 10^-10^ |
| *FABP3* | 3.87 | 1.5 x 10^-5^ |
| Top genes down-regulated | Fold-change | *P*-value |
| *LGR5* | -16.98 | 4.3 x 10^-19^ |
| *ANO4* | -9.34 | 1.3 x 10^-16^ |
| *VCAN* | -8.08 | 6.7 x 10^-9^ |
| *SFRP4* | -7.71 | 1.8 x 10^-8^ |
| *CYR61* | -6.98 | 5.5 x 10^-7^ |
| *GPC3* | -6.37 | 1.7 x 10^-10^ |
| *FOS* | -6.10 | 1.2 x 10^-8^ |
| *FOSB* | -6.01 | 1.3 x 10^-7^ |
| *EDNRA* | -5.67 | 1.0 x 10^-13^ |
| *TIPARP* | -5.46 | 5.0 x 10^-5^ |

**Table S6.** Top 10 genes up-regulated in zona glomerulosa (ZG) compared with zona fasciculata (ZF).

| ZG *vs.* ZF | Fold-change | *P*-value |
| --- | --- | --- |
| ***LGR5*** | 25.05 | 1.6 x 10^-23^ |
| ***VSNL1*** | 23.57 | 3.7 x 10^-23^ |
| ***ANO4*** | 19.89 | 6.6 x 10^-24^ |
| ***NEFM*** | 14.80 | 9.2 x 10^-12^ |
| ***VCAN*** | 14.34 | 1.9 x 10^-13^ |
| ***DACH1*** | 14.24 | 1.4 x 10^-21^ |
| ***NR4A2*** | 10.90 | 6.2 x 10^-8^ |
| *KIAA1210* | 8.90 | 5.4 x 10^-11^ |
| ***SFRP4*** | 8.67 | 2.3 x 10^-10^ |
| *C12orf75* | 7.22 | 4.4 x 10^-13^ |
| PG *vs.* PF | Fold-change | *P*-value |
| ***LGR5*** | 18.67 | 2.8 x 10^-12^ |
| ***VSNL1*** | 17.98 | 5.7 x 10^-12^ |
| ***NEFM*** | 14.09 | 6.2 x 10^-7^ |
| ***NR4A2*** | 12.33 | 1.0 x 10^-4^ |
| ***ANO4*** | 10.57 | 5.5 x 10^-11^ |
| ***VCAN*** | 10.51 | 3.6 x 10^-6^ |
| *OGN* | 9.98 | 7.2 x 10^-8^ |
| ***DACH1*** | 9.29 | 8.1 x 10^-11^ |
| *IGJ* | 8.32 | 3.4 x 10^-5^ |
| ***SFRP4*** | 7.98 | 1.2 x 10^-4^ |

*PG, zona glomerulosa samples from phaeochromocytoma patients only. PF, zona fasciculata samples from phaeochromocytoma patients only.

**Table S7.** Top 10 genes up-and down-regulated in aldsoterone-producing adenoma (APA) compared with zona glomerulosa (ZG) adjacent to a phaeochromocytoma.

| Top genes up-regulated | Fold-change | *P*-value |
| --- | --- | --- |
| ***SULT2A1*** | 6.53 | 5.0 x 10^-7^ |
| ***FABP3*** | 3.94 | 1.7 x 10^-3^ |
| *GSTA1* | 3.58 | 2.7 x 10^-2^ |
| ***MC2R*** | 3.29 | 4.5 x 10^-4^ |
| ***LRRC39*** | 2.97 | 3.4 x 10^-4^ |
| *IFITM10* | 2.95 | 2.2 x 10^-2^ |
| ***GSTA3*** | 2.93 | 1.3 x 10^-4^ |
| ***SLC37A2*** | 2.90 | 8.9 x 10^-3^ |
| *PAPSS2* | 2.87 | 2.9 x 10^-8^ |
| *CYP11A1* | 2.73 | 2.2 x 10^-5^ |
| Top genes down-regulated | Fold-change | *P*-value |
| ***LGR5*** | -6.54 | 1.2 x 10^-7^ |
| ***VCAN*** | -5.89 | 1.2 x 10^-4^ |
| ***CYR61*** | -5.47 | 4.3 x 10^-5^ |
| *NR4A2* | -4.39 | 8.7 x 10^-4^ |
| ***GPC3*** | -4.08 | 5.9 x 10^-9^ |
| *JCHAIN* | -3.94 | 4.9 x 10^-4^ |
| ***FOS*** | -3.87 | 1.5 x 10^-4^ |
| *ZFP36* | -3.66 | 5.0 x 10^-5^ |
| ***TIPARP*** | -3.58 | 2.6 x 10^-3^ |
| *ADAMTS1* | -3.47 | 1.3 x 10^-5^ |

**Table S8.** Top 5 Molecular and Cellular Functions enriched in aldosterone-producing adenoma (APA) compared with zona glomerulosa (ZG) adjacent to a phaeochromocytoma.

| Top APA Molecular and Cellular Functions | Molecules | *P*-value |
| --- | --- | --- |
| **Cellular Growth and Proliferation** | 81 | 8.0 x 10^-4^–4.2 x 10^-13^ |
| **Cellular Development** | 73 | 9.3 x 10^-4^–1.3 x 10^-11^ |
| **Cellular Movement** | 52 | 8.5 x 10^-4^–1.4 x 10^-10^ |
| **Cell Death and Survival** | 68 | 6.4 x 10^-4^–1.5 x 10^-10^ |
| **Lipid Metabolism** | 42 | 9.3 x 10^-4^–9.3 x 10^-10^ |

**Table S9.** Top 5 Canonical Pathways in aldosterone-producing adenoma (APA) samples compared to zona glomerulosa (ZG) samples adjacent to a phaeochromocytoma.

| Top Canonical Pathways | Overlap | *P*-value |
| --- | --- | --- |
| Hepatic Fibrosis / Hepatic Stellate Cell Activation | 8/183 | 3.5 x 10^-5^ |
| PXR/RXR Activation | 5/65 | 7.8 x 10^-5^ |
| **LPS/IL-1 Mediated Inhibition of RXR Function** | 8/219 | 1.3 x 10^-4^ |
| Aryl Hydrocarbon Receptor Signaling | 6/140 | 3.8 x 10^-4^ |
| Glutathione-mediated Detoxification | 3/29 | 9.7 x 10^-4^ |

**Table S10.** Genes associated with the Wnt Signaling Pathway that were differentially regulated in ZG (*vs.* zona fasciculata (ZF)) and in aldosterone-producing adenoma (APA) (compared to zona glomerulosa (ZG)).

| Gene symbol | Protein name | Fold-change (ZG *vs.* ZF) | *P*-value (ZG *vs.* ZF) | Fold-change (APA *vs.* ZG) | *P*-value (APA *vs.* ZG) |
| --- | --- | --- | --- | --- | --- |
| *LGR5* | Leucine-rich repeat-containing G-protein coupled receptor 5 | 25.05 | 1.57 x 10^-23^ | -16.98 | 4.31 x 10^-19^ |
| *SFRP4* | Secreted frizzled-related protein 4 | 8.67 | 2.30 x 10^-10^ | -7.71 | 1.82 x 10^-8^ |
| *LEF1* | Lymphoid enhancer-binding factor 1 | 5.88 | 4.29 x 10^-15^ | n.s. |  |
| *BICC1* | Protein bicaudal C homolog 1 | 4.65 | 5.28 x 10^-11^ | -5.13 | 2.11 x 10^-10^ |
| *FRZB* | Secreted frizzled-related protein 3 | 3.55 | 2.40 x 10^-8^ | -2.81 | 1.33 x 10^-5^ |
| *APCDD1* | Protein APCDD1 | 3.51 | 2.61 x 10^-14^ | n.s. |  |
| *DCDC2* | Doublecortin domain-containing protein 2 | 3.21 | 7.87 x 10^-15^ | -3.04 | 1.79 x 10^-12^ |
| *BAMBI* | BMP and activin membrane-bound inhibitor homolog | 2.75 | 2.16 x 10^-4^ | -3.28 | 1.12 x 10^-4^ |
| *FZD6* | Frizzled-6 | 2.69 | 9.36 x 10^-11^ | -2.17 | 6.38 x 10^-7^ |
| *SULF2* | Extracellular sulfatase Sulf-2 | 2.62 | 3.27 x 10^-11^ | n.s. |  |
| *SNAI2* | Zinc finger protein SNAI2 | 2.40 | 7.88 x 10^-7^ | -2.43 | 5.07 x 10^-6^ |
| *WNT4* | Protein Wnt-4 | 2.25 | 3.21 x 10^-8^ | n.s. |  |
| *CAV1* | Caveolin-1 | 2.16 | 1.58 x 10^-5^ | n.s. |  |
| *DKK3* | Dickkopf-related protein 3 | 2.09 | 4.65 x 10^-10^ | n.s. |  |
| *GRB10* | Growth factor receptor-bound protein 10 | -2.01 | 3.33 x 10^-6^ | n.s. |  |

n.s., not significant.

**Table S11.** Genes associated with the Wnt signaling pathway that were differentially regulated in zona glomerulosa (ZG) (*vs.* zona fasciculata (ZF)) adjacent to a phaeochromocytoma.

| Gene symbol | Protein name | Fold-change  (PG *vs.* PF) | *P*-value  (PG *vs.* PF) |
| --- | --- | --- | --- |
| *LGR5* | Leucine-rich repeat-containing G-protein coupled receptor 5 | 18.67 | 2.76 x 10^-12^ |
| *SFRP4* | Secreted frizzled-related protein 4 | 7.98 | 1.22 x 10^-4^ |
| *LEF1* | Lymphoid enhancer-binding factor 1 | 5.47 | 1.30 x 10^-7^ |
| *APCDD1* | Protein APCDD1 | 4.10 | 1.36 x 10^-9^ |
| *FRZB* | Secreted frizzled-related protein 3 | 3.38 | 9.03 x 10^-4^ |
| *BICC1* | Protein bicaudal C homolog 1 | 3.29 | 1.05 x 10^-3^ |
| *SNAI2* | Zinc finger protein SNAI2 | 2.71 | 5.28 x 10^-4^ |
| *FZD6* | Frizzled-6 | 2.49 | 1.94 x 10^-4^ |
| *DKK3* | Dickkopf-related protein 3 | 2.48 | 6.81 x 10^-8^ |
| *DCDC2* | Doublecortin domain-containing protein 2 | 2.35 | 4.33 x 10^-5^ |
| *ITGA3* | Integrin alpha-3 | 2.06 | 1.35 x 10^-3^ |
| *GRB10* | Growth factor receptor-bound protein 10 | -2.14 | 1.93 x 10^-3^ |

*PG, ZG samples from phaeochromocytoma patients only. PF, ZF samples from phaeochromocytoma patients only.

**Table S12.** Genes associated with steroid, organic hydroxy compound, alcohol related processes that were differentially regulated in zona glomerulosa (ZG) (*vs.* zona fasciculata (ZF)).

| Gene symbol | Protein name | Fold-change  (PG *vs.* PF) | *P*-value  (PG *vs.* PF) | Fold-change  (ZG *vs.*ZF) | *P*-value  (ZG *vs.*ZF) |
| --- | --- | --- | --- | --- | --- |
| *CYP3A4* | Cytochrome P450 3A4 | 3.98 | 4.76 x 10^-5^ | 3.10 | 4.93 x 10^-8^ |
| *CYP11B2* | Cytochrome P450 11B2 | 2.57 | 2.32 x 10^-2^ | n.s. |  |
| *RBP4* | Retinol-binding protein 4 | 2.48 | 4.98 x 10^-2^ | 5.03 | 3.79 x 10^-7^ |
| *CYP1B1* | Cytochrome P450 1B1 | 2.42 | 7.61 x 10^-3^ | 2.17 | 3.86 x 10^-5^ |
| *CYP39A1* | 24-hydroxycholesterol 7-alpha-hydroxylase | 2.33 | 7.39 x 10^-5^ | n.s. |  |
| *PLA2G1B* | Phospholipase A2 | 2.20 | 1.05 x 10^-2^ | n.s. |  |
| *SORD* | Sorbitol dehydrogenase | -2.01 | 3.09 x 10^-2^ | -2.53 | 1.05 x 10^-4^ |
| *DHCR24* | Delta(24)-sterol reductase | -2.12 | 4.73 x 10^-6^ | -2.34 | 1.08 x 10^-13^ |
| *SULT1E1* | Estrogen sulfotransferase | -2.16 | 1.91 x 10^-4^ | n.s. |  |
| *DHCR7* | 7-dehydrocholesterol reductase | -2.41 | 1.02 x 10^-3^ | -2.72 | 9.45 x 10^-8^ |
| *APOE* | Apolipoprotein E | -2.84 | 5.51 x 10^-4^ | -3.05 | 1.01 x 10^-7^ |
| *CYP17A1* | Steroid 17-alpha-hydroxylase/17,20 lyase | -3.18 | 4.92 x 10^-4^ | -3.34 | 1.27 x 10^-8^ |
| *PON1* | Serum paraoxonase/arylesterase 1 | -3.56 | 1.78 x 10^-2^ | -2.64 | 1.53 x 10^-3^ |
| *CYP11A1* | Cholesterol side-chain cleavage enzyme | -3.68 | 1.56 x 10^-7^ | -4.31 | 5.39 x 10^-16^ |

*PG, zona glomerulosa (ZG) samples from phaeochromocytoma patients only. PF, zona fasciculate (ZF) samples from phaeochromocytoma patients only. n.s., not significant.

**Table S13.** Gene Ontology (GO) of biological processes >5 fold-enriched in zona glomerulosa (ZG) compared with zona fasciculata (ZF) samples from phaeochromocytoma patients.

| GO biological processes | *P*-value |
| --- | --- |
| Steroid biosynthetic process | 4.24 x 10^-2^ |
| Steroid metabolic process | 2.34 x 10^-3^ |
| Negative regulation of Wnt signaling pathway | 1.98 x 10^-2^ |
| Alcohol metabolic process | 5.23 x 10^-3^ |
| Organic hydroxy compound metabolic process | 9.57 x 10^-3^ |
| Regulation of Wnt signaling pathway | 1.65 x 10^-2^ |

**Table S14.** Top 5 Canonical Pathways in zona glomerulosa (ZG) samples compared to zona fasciculata (ZF) samples adjacent to a phaeochromocytoma.

| Top Canonical Pathways | Overlap | *P*-value |
| --- | --- | --- |
| **LPS/IL-1 Mediated Inhibition of RXR Function** | 21/219 | 2.5 x 10^-10^ |
| FXR/RXR Activation | 13/127 | 3.2 x 10^-7^ |
| Cholesterol Biosynthesis I | 5/13 | 1.7 x 10^-6^ |
| Cholesterol Biosynthesis II (via 24,25-dihydrolanosterol) | 5/13 | 1.7 x 10^-6^ |
| Cholesterol Biosynthesis III (via Desmosterol) | 5/13 | 1.7 x 10^-6^ |

**Table S15.** Genes associated with the ‘NRF2-mediated Oxidative Stress Response’ that were differentially regulated in zona glomerulosa (ZG) (*vs.* zona fasciculata (ZF)) and in aldosterone-producing adenoma (APA) (*vs.* ZG).

| Gene symbol | Protein name | Fold-change (ZG *vs.* ZF) | *P*-value (ZG *vs.* ZF) | Fold-change (APA *vs.* ZG) | *P*-value (APA *vs.* ZG) |
| --- | --- | --- | --- | --- | --- |
| *AOX1* | Aldehyde Oxidase 1 | -2.21 | 1.53 x 10^-4^ | n.s. |  |
| *CYP3A4* | Cytochrome P450, Family 3, Subfamily A, Polypeptide 4 | 3.10 | 4.93 x 10^-8^ | -3.38 | 1.10 x 10^-7^ |
| *CYP3A5* | Cytochrome P450, Family 3, Subfamily A, Polypeptide 5 | n.s. |  | -2.46 | 2.04 x 10^-4^ |
| *FOS* | FBJ Murine Osteosarcoma Viral Oncogene Homolog | 2.75 | 9.63 x 10^-5^ | -6.01 | 1.34 x 10^-7^ |
| *FOSB* | FBJ Murine Osteosarcoma Viral Oncogene Homolog B | n.s. |  | -6.10 | 1.18 x 10^-8^ |
| *GSTA1* | Glutathione S-Transferase Alpha 1 | -20.75 | 1.02 x 10^-10^ | n.s. |  |
| *GSTA3* | Glutathione S-Transferase Alpha 3 | -8.64 | 2.10 x 10^-15^ | 4.01 | 3.81 x 10^-8^ |
| *GSTA4* | Glutathione S-Transferase Alpha 4 | -2.47 | 7.76 x 10^-12^ | n.s. |  |
| *GSTM4* | Glutathione S-Transferase Mu 4 | 2.25 | 1.38 x 10^-3^ | n.s. |  |
| *JUN* | Jun Proto-Oncogene | n.s. |  | -2.55 | 3.87 x 10^-7^ |
| *JUNB* | Jun B Proto-Oncogene | 2.89 | 5.62 x 10^-6^ | -3.38 | 3.26 x 10^-6^ |
| *MAP3K5* | Mitogen-Activated Protein Kinase Kinase Kinase 5 | -2.13 | 9.49 x 10^-9^ | n.s. |  |
| *MGST1* | Microsomal Glutathione S-Transferase 1 | -2.28 | 7.18 x 10^-11^ | n.s. |  |
| *PIK3R1* | Phosphoinositide-3-Kinase, Regulatory Subunit 1 (Alpha) | 2.04 | 1.59 x 10^-5^ | -2.27 | 9.85 x 10^-6^ |
| *PRKCA* | Protein Kinase C, Alpha | -4.12 | 2.06 x 10^-13^ | n.s. |  |
| *PRKCH* | Protein Kinase C, Eta | n.s. |  | 3.51 | 7.83 x 10^-8^ |
| *SCARB1* | Scavenger Receptor Class B, Member 1 | -3.04 | 3.46 x 10^-14^ | 2.56 | 2.51 x 10^-10^ |

*n.s., not significant.

**Table S16.** Genes associated with the ‘LPS/IL-1 Mediated Inhibition of RXR Function’ that were differentially regulated in zona glomerulosa (ZG) (vs. zona fasciculata (ZF)) and in aldosterone-producing adenoma (APA) (compared with ZG).

| Gene symbol | Protein name | Fold-change  (ZG *vs.* ZF) | *P*-value  (ZG *vs.* ZF) | Fold-change  (APA *vs.* ZG) | *P*-value  (APA *vs.* ZG) |
| --- | --- | --- | --- | --- | --- |
| *ABCA1* | ATP Binding Cassette Subfamily A Member 1 | -2.39 | 3.65 x 10^-13^ | 2.05 | 3.07 x 10^-9^ |
| *ABCB1* | ATP Binding Cassette Subfamily B Member 1 | -2.65 | 7.46 x 10^-14^ | n.s. |  |
| *ACSL1* | Acyl-CoA Synthetase Long-Chain Family Member 1 | -2.43 | 3.49 x 10^-6^ | 2.86 | 1.11 x 10^-6^ |
| *ACSF2* | Acyl-CoA Synthetase Family Member 2 | n.s. |  | 2.23 | 1.83 x 10^-7^ |
| *APOC1* | Apolipoprotein C-I | -3.39 | 5.34 x 10^-7^ | n.s. |  |
| *APOE* | Apolipoprotein E | -3.05 | 1.01 x 10^-7^ | 2.13 | 4.45 x 10^-4^ |
| *FABP3* | Fatty Acid Binding Protein 3, Muscle And Heart | -2.48 | 7.82 x 10^-4^ | 3.87 | 1.52 x 10^-5^ |
| *FABP6* | Fatty Acid Binding Protein 6, Ileal | -3.05 | 6.70 x 10^-7^ | n.s. |  |
| *NR0B2* | Nuclear Receptor Subfamily 0, Group B, Member 2 | -4.73 | 1.02 x 10^-11^ | n.s. |  |
| *NR1H4* | Nuclear Receptor Subfamily 1, Group H, Member 4 | -2.38 | 1.68 x 10^-9^ | n.s. |  |
| *PAPSS2* | 3'-Phosphoadenosine 5'-Phosphosulfate Synthase 2 | -5.22 | 1.07 x 10^-20^ | 3.73 | 6.66 x 10^-15^ |
| *SREBF1* | Sterol Regulatory Element Binding Transcription Factor 1 | -2.20 | 4.77 x 10^-10^ | n.s. |  |
| *SULT2A1* | Sulfotransferase Family 2A Member 1 | -8.21 | 4.50 x 10^-16^ | 11.08 | 1.79 x 10^-16^ |
| *UST* | Uronyl-2-Sulfotransferase | 2.72 | 5.90 x 10^-12^ | n.s. |  |

*The genes *MGST1*, *SCARB1*, *GSTA1*, *GSTA3*, *GSTA4*, *GSTM4*, *CYP3A4*, *CYP3A5* are also associated with the ‘LPS/IL-1 Mediated Inhibition of RXR Function’ pathway. Fold-change and *P*-values in ZG *vs.* ZF and APA *vs.* ZG are shown in Table S15. n.s., not significant.

**Table S17.** Top 10 genes differentially regulated in *KCNJ5* mutant aldsoterone-producing adenoma (APA) compared with wild-type.

| Top genes up-regulated | Fold-change | *P*-value |
| --- | --- | --- |
| *ACSS3* | 5.45 | 4.20 x 10^-5^ |
| *SCARNA9L* | 5.19 | 1.85 x 10^-2^ |
| *HTR2B* | 4.10 | 2.97 x 10^-6^ |
| *SNORD32A* | 3.68 | 2.69 x 10^-2^ |
| *KLHL4* | 3.62 | 9.88 x 10^-4^ |
| *TNFSF10* | 3.52 | 2.59 x 10^-3^ |
| *CST2* | 3.12 | 5.09 x 10^-7^ |
| *CFI* | 2.94 | 1.51 x 10^-2^ |
| *FCGR3A* | 2.91 | 3.58 x 10^-2^ |
| *LPHN3* | 2.88 | 4.26 x 10^-3^ |
| Top genes down-regulated | Fold-change | *P*-value |
| *PTPRZ1* | -3.04 | 4.75 x 10^-5^ |
| *EDNRB* | -3.11 | 8.88 x 10^-5^ |
| *C11orf41* | -3.21 | 5.07 x 10^-4^ |
| *NETO2* | -3.40 | 1.40 x 10^-3^ |
| *VPREB3* | -3.60 | 2.73 x 10^-2^ |
| *CA2* | -3.95 | 1.49 x 10^-3^ |
| *NEFM* | -3.96 | 4.35 x 10^-3^ |
| *SLC35F1* | -4.14 | 1.84 x 10^-5^ |
| *BEX1* | -5.23 | 5.98 x 10^-4^ |
| *MYOM1* | -5.49 | 2.08 x 10^-5^ |

**Table S18.** Top 10 genes differentially regulated in zona glomerulosa (ZG) from *KCNJ5* mutant patient compared with ZG from wild-type patient.

| Top genes up-regulated | Fold-change | *P*-value |
| --- | --- | --- |
| *NMI* | 3.93 | 9.73 x 10^-5^ |
| *RRP7B* | 3.93 | 2.35 x 10^-2^ |
| *SNORD78* | 3.86 | 2.53 x 10^-2^ |
| *SNORD32A* | 3.73 | 3.15 x 10^-2^ |
| *SNORD28* | 3.57 | 3.46 x 10^-2^ |
| *ATXN7L1* | 3.23 | 4.07 x 10^-2^ |
| *ZNF277* | 3.23 | 9.98 x 10^-3^ |
| *DDAH1* | 2.99 | 9.83 x 10^-5^ |
| *SNORA22* | 2.95 | 2.96 x 10^-2^ |
| *LOC400986* | 2.57 | 2.26 x 10^-2^ |
| Top genes down-regulated | Fold-change | *P*-value |
| *MYH11* | -2.26 | 3.40 x 10^-3^ |
| *C4orf46* | -2.37 | 4.23 x 10^-2^ |
| *CA2* | -2.40 | 4.44 x 10^-2^ |
| *SORD* | -2.40 | 1.01 x 10^-2^ |
| *MIR323* | -2.43 | 2.38 x 10^-2^ |
| *SMOC2* | -2.47 | 4.54 x 10^-5^ |
| *TNXA* | -2.49 | 2.88 x 10^-2^ |
| *SNORA45* | -2.84 | 1.98 x 10^-2^ |
| *RNF185* | -3.12 | 4.73 x 10^-2^ |
| *C6orf52* | -3.57 | 1.62 x 10^-4^ |

**Table S19.** Top 10 genes differentially regulated in zona fasciculata (ZF) from *KCNJ5* mutant patient compared with ZF from wild-type patient.

| Top genes up-regulated | Fold-change | *P*-value |
| --- | --- | --- |
| *NR4A2* | 4.71 | 1.17 x 10^-2^ |
| *SNORD76* | 4.29 | 6.86 x 10^-3^ |
| *TIPARP* | 3.66 | 1.83 x 10^-2^ |
| *SNORD63* | 3.58 | 1.67 x 10^-2^ |
| *FOS* | 3.19 | 1.42 x 10^-3^ |
| *MCM9* | 2.89 | 8.62 x 10^-3^ |
| *SNORA7B* | 2.72 | 4.10 x 10^-2^ |
| *IL7R* | 2.60 | 1.68 x 10^-2^ |
| *APOD* | 2.53 | 2.47 x 10^-2^ |
| *SGK1* | 2.52 | 9.75 x 10^-3^ |
| Top genes down-regulated | Fold-change | *P*-value |
| *DEFB109P1B* | -2.19 | 3.51 x 10^-2^ |
| *NUDT13* | -2.20 | 1.60 x 10^-3^ |
| *KRT18* | -2.23 | 3.55 x 10^-2^ |
| *SNORD45B* | -2.74 | 2.48 x 10^-3^ |
| *ZDBF2* | -2.78 | 3.98 x 10^-2^ |
| *C2orf66* | -2.79 | 3.70 x 10^-3^ |
| *DEXI* | -2.84 | 2.54 x 10^-2^ |
| *LOC100507404* | -2.98 | 3.89 x 10^-3^ |
| *SNORD38A* | -3.03 | 4.86 x 10^-2^ |
| *LRRFIP1* | -3.44 | 2.38 x 10^-2^ |

**Table S20.** Top 5 molecular and cell functions of differentially expressed genes in *KCNJ5* mutant samples compared with *KCNJ5* wild-type samples.

| *KCNJ5* mutant APA *vs.* wild-type | | |
| --- | --- | --- |
| Top Molecular and Cell functions | Molecules | *P*-value |
| Cell Morphology | 36 | 1.16 x 10^-2^ - 4.11 x 10^-5^ |
| Cell Death and Survival | 49 | 1.16 x 10^-2^ - 5.04 x 10^-5^ |
| Cell-To-Cell Signaling and Interaction | 29 | 1.16 x 10^-2^ - 9.99 x 10^-5^ |
| Cellular Assembly and Organization | 25 | 1.16 x 10^-2^ - 9.99 x 10^-5^ |
| Cellular Development | 34 | 1.16 x 10^-2^ - 1.35 x 10^-4^ |
| ZG adjacent to *KCNJ5* mutant APA *vs.* wild-type | | |
| Top Molecular and Cell functions | Molecules | *P*-value |
| Carbohydrate Metabolism | 2 | 7.26 x 10^-3^ - 1.82 x 10^-3^ |
| Cellular Development | 5 | 4.46 x 10^-2^ - 1.82 x 10^-3^ |
| Cellular Function and Maintenance | 9 | 4.98 x 10^-2^ - 1.82 x 10^-3^ |
| Cellular Movement | 5 | 4.98 x 10^-2^ - 1.82 x 10^-3^ |
| Drug Metabolism | 1 | 3.58 x 10^-2^ - 1.82 x 10^-3^ |
| ZF adjacent to *KCNJ5* mutant APA *vs.* wild-type | | |
| Top Molecular and Cell functions | Molecules | *P*-value |
| Cell Cycle | 14 | 1.23 x 10^-2^ - 1.25 x 10^-5^ |
| Cellular Development | 21 | 1.64 x 10^-2^ - 1.25 x 10^-5^ |
| Cell Death and Survival | 20 | 1.64 x 10^-2^ - 1.31 x 10^-5^ |
| Cellular Growth and Proliferation | 20 | 1.64 x 10^-2^ - 3.25 x 10^-5^ |
| Cellular Compromise | 10 | 1.64 x 10^-2^ - 1.81 x 10^-4^ |

**Table S21.** Top 5 canonical pathways of differentially expressed genes in *KCNJ5* mutant samples compared with *KCNJ5* wild-type samples.

| *KCNJ5* mutant APA *vs.* wild-type | | |
| --- | --- | --- |
| Top Canonical Pathways | Overlap | *P*-value |
| Breast Cancer Regulation by Stathmin1 | 2.6 % (5/191) | 5.18 x 10^-3^ |
| Glycolysis I | 8.0 % (2/25) | 9.19 x 10^-3^ |
| Gluconeogenesis I | 8.0 % (2/25) | 9.19 x 10^-3^ |
| Gap Junction Signaling | 2.6 % (4/155) | 1.28 x 10^-2^ |
| Catecholamine Biosynthesis | 25.0 % (1/4) | 2.30 x 10^-2^ |
| ZG adjacent to *KCNJ5* mutant APA *vs.* wild-type | | |
| Top Canonical Pathways | Overlap | *P*-value |
| Sorbitol Degradation I | 100.0 % (1/1) | 1.82 x 10^-3^ |
| Histamine Degradation | 7.7 % (1/13) | 2.34 x 10^-2^ |
| Fatty Acid -oxidation | 6.2 % (1/16) | 2.87 x 10^-2^ |
| Oxidative Ethanol Degradation III | 6.2 % (1/16 | 2.87 x 10^-2^ |
| Putrescine Degradation III | 5.9 % 1/17 | 3.05 x 10^-2^ |
| ZF adjacent to *KCNJ5* mutant APA *vs.* wild-type | | |
| Top Canonical Pathways | Overlap | *P*-value |
| Glucocorticoid Receptor Signaling | 1.5 % (4/275) | 2.45 x 10^-3^ |
| ERK5 Signaling | 3.2 % (2/63) | 7.52 x 10^-3^ |
| Nicotine Degradation II | 3.2 % (2/63) | 7.52 x 10^-3^ |
| UDP-N-acetyl-D-glucosamine Biosynthesis II | 16.7 % (1/6) | 1.23 x 10^-2^ |
| UVA-Induced MAPK Signaling | 2.3 % (2/88) | 1.43 x 10^-2^ |

**Figure S1.** Heterogenous CYP11B2 expression in aldosterone-producing adenomas (APA). (A and B) *KCNJ5* mutant APA tissue, boxed areas in the 4× graphs (A) are shown in the corresponding 20× graphs (B); (C and D) KCNJ5 wild-type APA tissue, boxed areas in the 4× graphs (C) are shown in the corresponding 20× graphs (D). n=8.

**Figure S2.** Confirmation of zona glomerulosa (ZG) and zona fasciculata (ZF) steroid expression profile in ZF and ZG samples. qPCR of (A) *CYP11B2* mRNA expression as a hallmark gene of ZG, and (B) *CYP11B1* and (C) *CYP17A1* mRNA expression as hallmark genes of ZF. n=21.
